# Single-cell transcriptomic profiling reveals specific maturation signatures in human cardiomyocytes derived from *LMNB2*-inactivated induced pluripotent stem cells

**DOI:** 10.3389/fcell.2022.895162

**Published:** 2022-11-28

**Authors:** Jie Wang, William Morgan, Ankur Saini, Tao Liu, John Lough, Lu Han

**Affiliations:** ^1^ Department of Biostatistics and Bioinformatics, Roswell Park Comprehensive Cancer Center, Buffalo, NY, United States; ^2^ Department of Pediatrics, Medical College of Wisconsin, Milwaukee, WI, United States; ^3^ Division of Pediatric Cardiology, Herma Heart Institute, Children’s Hospital of Wisconsin, Milwaukee, WI, United States; ^4^ Cardiovascular Center, Medical College of Wisconsin, Milwaukee, WI, United States; ^5^ Department of Cell Biology, Neurobiology and Anatomy, Medical College of Wisconsin, Milwaukee, WI, United States

**Keywords:** cardiomyocyte maturation, human iPSCs, single-cell RNA-seq, Lmnb2, proliferation, human fetal heart, lamin B2

## Abstract

Mammalian cardiomyocyte maturation entails phenotypic and functional optimization during the late fetal and postnatal phases of heart development, both processes driven and coordinated by complex gene regulatory networks. Cardiomyocytes derived from human induced pluripotent stem cells (iPSCs) are heterogenous and immature, barely resembling their adult *in vivo* counterparts. To characterize relevant developmental programs and maturation states during human iPSC-cardiomyocyte differentiation, we performed single-cell transcriptomic sequencing, which revealed six cardiomyocyte subpopulations, whose heterogeneity was defined by cell cycle and maturation states. Two of those subpopulations were characterized by a mature, non-proliferative transcriptional profile. To further investigate the proliferation-maturation transition in cardiomyocytes, we induced loss-of-function of *LMNB2*, which represses cell cycle progression in primary cardiomyocytes *in vivo*. This resulted in increased maturation in *LMNB2*-inactivated cardiomyocytes, characterized by transcriptional profiles related to myofibril structure and energy metabolism. Furthermore, we identified maturation signatures and maturational trajectories unique for control and *LMNB2*-inactivated cardiomyocytes. By comparing these datasets with single-cell transcriptomes of human fetal hearts, we were able to define spatiotemporal maturation states in human iPSC-cardiomyocytes. Our results provide an integrated approach for comparing *in vitro*-differentiated cardiomyocytes with their *in vivo* counterparts and suggest a strategy to promote cardiomyocyte maturation.

## Introduction

Heart development is governed by complex stage-specific transcriptional regulation that coordinates cardiac cell specification, tissue morphogenesis, and functional maturation. The ability to generate cardiomyocytes (CMs) derived from human induced pluripotent stem cells (iPSCs) has allowed us to gain a better understanding of heart development and related diseases (Murry and Keller, 2008). Presently, iPSC-CMs hold great promise for translational applications including heart regeneration, disease modeling, and drug screening (Laflamme and Murry, 2011; Han et al., 2014; Shi et al., 2017; Tzahor and Poss, 2017). However, although current differentiation methods can generate large quantities of purified cardiomyocytes, these cells do not resemble their adult *in vivo* counterparts in terms of morphology, function, and transcriptional profiles (Doss et al., 2012; Lundy et al., 2013; Chong et al., 2014; Scuderi and Butcher, 2017; Ronaldson-Bouchard et al., 2018). Hence, novel research into cardiac development and application of the subsequent findings to design strategies for promoting full maturation of human iPSC-CMs is needed to circumvent this barrier. Recent advances in single-cell technology have allowed us to define human iPSC-CMs at exceedingly high resolution. For example, single-cell RNA analyses of *in vivo* human and mouse heart tissue have revealed highly detailed transcriptional profiles and novel features of cellular heterogeneity during cardiomyocyte specification and maturation (DeLaughter et al., 2016; Li et al., 2016; Cui et al., 2019; de Soysa et al., 2019; Litvinukova et al., 2020; Wang et al., 2020). Study of transcriptional dynamics has also yielded novel knowledge regarding spatiotemporal relationships between human heart cells during cardiac development *in vivo* (Asp et al., 2019). Moreover, transcriptional regulators promoting specific aspects of cardiomyocyte maturation have been identified (Churko et al., 2018; Friedman et al., 2018). However, both cellular heterogeneity among human iPSC-CMs and the extent to which these cardiomyocytes recapitulate transcriptional profiles of their *in vivo* counterparts are poorly understood. In addition, although cardiomyocyte maturation is assumed to be inversely related to the process of proliferation, there are few lines of evidence to support direct links between proliferation and maturation, and the transcriptional profiles accompanying this transition remain unknown (Chattergoon et al., 2012; Hirose et al., 2019; D'Uva et al., 2015).

To investigate cellular heterogeneity during cardiomyocyte differentiation, we first characterized the single-cell transcriptome of human iPSC-CMs induced by modulating WNT signaling, and we report here our findings identifying cellular subpopulations and distinct developmental gene profiles that define cardiomyocyte maturity. Then, we focused on the exit from the cell cycle as a phenotypic hallmark of cardiomyocyte maturation (Mollova et al., 2013; Senyo et al., 2013), in which regard we have recently identified a key regulator, the nuclear lamina gene *LMNB2* (encoding lamin B2 protein) (Han et al., 2020). In this study, disruption of the *LMNB2* gene revealed an accelerated process of cardiomyocyte maturation. In addition, using unsupervised analysis we uncovered developmental programs involved in the different phases of cardiomyocyte maturation. By comparing our own results with the single-cell RNA profiles of human fetal heart tissue, we were able to define developmental similarities between human iPSC-CMs and their *in vivo* counterparts. Although human iPSC-CMs are relatively naïve, our data reveal that they were able to continually mature, thus enabling the development of strategies to promote maturation *in vitro*. The integrative platform to compare single-cell transcriptional profiles that we have established constitutes a valuable way of integrating public datasets from different consortia and will allow faster advances in understanding and controlling cardiomyocyte maturation.

## Materials and methods

### Human pluripotent stem cell cultures

CiPS001-13 iPSCs generously donated by Drs. Kevin Bersell and Dan Roden (Vanderbilt University) were maintained in mTESR one medium (StemTechnologies 85850) supplemented with 1% Antibiotic-Antimycotic (Life Technologies 15240062) on BD hESC-qualified Matrigel (Corning 354277). Cells were passaged every 5–6 days using ReLeSR dissociation reagent (Stem Cell Technologies 5872) according to the manufacturer’s protocol. Then, 10 mM of Rho-associated kinase inhibitor (ROCK) Y27632 (EMD 68000) was added to the medium for the first 24 h after passaging. Human iPSCs were incubated at 37°C in 5% CO_2_ and maintained in 2 ml of mTESR one medium with changes every 48 h until the day of passage.

### CRISPR-Cas9 knockout of *LMNB2* in iPSCs

The Cas9 plasmid was engineered with green fluorescent protein (GFP) fused into lentiCRISPRv2 (Addgene 52961), which was generously shared by Dr. Yi Sheng (University of Pittsburgh). The iPSC line CiPS001-013 was transfected with CRISPR plasmid-carrying guide RNA oligos (5′-CACCG AGA​CGG​CTC​GAG​AGC​GTG​CC-3’; 5′-AAA​CGG​CAC​GCT​CTC​GAG​CCG​TCT​C- 3′) targeting the chr19:2,444,459-2,444,479 plus strand, a region on exon2 of the human *LMNB2* gene. Single cells transiently expressing GFP were isolated by flow cytometry. Indels were detected using SURVEYOR nuclease assays (Transgenomic 706025) (Ran et al., 2013). A SURVEYOR PCR primer pair was designed to amplify a 451 bp band containing the targeted region of exon 2. Using SURVEYOR nuclease digestion, heterozygous mutations were detected in two clones. DNA from the remaining clones was mixed with DNA from wild type iPSCs, denatured, annealed, and digested with SURVEYOR nuclease. Several clones were identified containing homozygous mutations. Results were confirmed by Sanger DNA sequencing, revealing one base pair thymidine insertion, causing frame-shift insertion in clones such as C35 and C103. The characterization results were reported in our previous publication (Han et al., 2020). To determine potential off-target activity of the gRNAs we followed instructions in the original paper (Ran et al., 2013). We used the recommended online CRISPR Design Tool (http://crispr.mit.edu) to predict genomic off-target sites and examined the potential off-target sites with scores >0. All predicted exonic homologous sites were verified by DNA Sanger sequencing. No off-target mutations were observed at any site.

### Cardiomyocyte differentiation of human iPSCs

At all pluripotent and differentiative stages, cells were maintained at 37 °C in 5% CO_2_. CiPS001-13 human iPSCs were split using ReLeSR dissociation reagent and seeded onto 12-well plates coated with growth-factor-reduced (GFR) Matrigel (Corning 356230). Cells were expanded in mTESR one medium (StemTechnologies 85850) for 4 days, during which time the medium was replaced at 24 and 72 h and allowed to reach 80–90% confluency. On differentiation-day 0 (subsequently denoted d0), differentiation was induced by exchanging the medium for RPMI 1640 containing 2% B-27 without insulin (subsequently RPMI/B27-; Life Technologies 11875–093, A1895601) including 1% Antibiotic-Antimycotic (Life Technologies 15240062) and supplemented with 12 mM GSK3 inhibitor CHIR99021 (Selleck S2924) to induce differentiation. After precisely 24 h, medium was replaced with 2 ml fresh RPMI/B27- without CHIR and the cells were expanded for an additional 48 h. On d3, medium was replaced with 2 ml fresh RPMI/B27- containing 5 mM WNT inhibitor IWR-1 (Sigma I0161) and incubated for 48 h. On d5, medium was exchanged for 2 ml of the same medium without IWR-1, followed on d7 by changing to RPMI/B27 with insulin (ie RPMI/B27+, Life Technologies 17504044). On d9, contracting cells appeared. To enrich the cardiomyocyte population, on d12 medium was exchanged for RPMI/B27 + without glucose (Life Technology 11879-020) containing 5 mM sodium d-lactate.

### Sub-culturing human iPSC-CMs

On d15, cells were re-plated onto GFR Matrigel (Corning)-coated wells as follows. Cells were rinsed briefly with phosphate buffered saline (PBS), followed by addition of trypsin (TrypLE; Life Technology 12605010) and incubation for 10–12 min at 37°C. Cells were then suspended by gentle pipetting, followed by adding an equal volume of stop medium [50% DMEM (Life technology 11965092); 50% Heat Inactivated FBS (Life Technology 10437028); 10 mg/ml DNase (Roche 10104159001)] to inactivate TrypLE. Cells were gently resuspended, filtered through a 70-µm nylon cell strainer (Fisher 352350) and spun at 800 rpm for 5 min at room temperature. Cells were counted and replated at 100-150k/cm^2^ in RPMI/B27+ with lactate until the day of analysis designated in the Text and Figures.

### Single-cell library preparation and sequencing

Single cardiomyocytes were prepared by dissociating the cells in ReLeSR (Stem Cell Technologies) according to the manufacturer’s protocol. The cell suspensions were filtered through a 70-µm Nylon cell strainer (Fisher), centrifuged, washed in PBS/0.04% BSA and diluted to 1,000 cells/µL. Single-cell libraries were prepared according to the 10X Chromium 3′ reagent kits v2 user guide and sequenced on the Illumina Hiseq 4000 platform. Each sample targeted 500 cells at a read depth of 100K reads per cell.

### Single-cell transcriptomic analysis

Raw sequencing data were converted to FastQ format, aligned to human reference genome (version hg38 assembly from UCSC), and quantified using the Cell Ranger from 10x Genomics. Cells with fraction of mitochondrial RNAs higher than 50% and cells with low complexity expressing few unique genes (2 Mean absolute deviation (MAD) below the mean) were filtered. Genes that expressed in more than 5 cells were retained for further analysis. The Seurat R package was utilized to perform the downstream analysis. The data were normalized using the LogNormalize method and then analyzed to identify top 2,000 highly variable genes, which were used to perform principal component analysis (PCA). A K-nearest neighbor (KNN) graph was constructed based on PCA space using FindNeighbors function with top 20 PCs. FindClusters function was applied to cluster cells using Louvain algorithm with resolution set to 0.5. To visualize the data, non-linear dimensional reduction method Uniform Manifold Approximation and Projection (UMAP) were performed. For all differential expression, a Wilcoxon Rank Sum test was used to compare between two groups of cells. Enriched gene pathways and Gene Ontology analysis were identified using EnrichR package (Xie et al., 2021).

### RNA velocity analysis

The mapping data from the scRNA-seq were processed using the velocyto package to obtain two count matrices of nascent (unspliced) and mature (spliced) mRNA abundance. The scVelo package was used to perform the RNA velocity analysis. The filtered and normalized data generated from Seurat were used to apply the dynamical model to solve the full transcriptional dynamics. The velocities were projected to the pre-computed UMAP embedding and annotated clusters. Driver genes were detected by high likelihoods in the dynamic model.

### Pseudotime analysis

When performing the pseudotime analysis, the C1 cluster was removed to avoid the effect from the non-cardiomyocytes. Pseudotime analysis was done with Monocle2 on the UMI count data. Variable genes detected by Seurat were used to construct trajectories. To identify the genes associated with the pseudotime, additive models of gene expression were fitted as a function of pseudotime, and significant genes with false discovery rate (FDR) less than 10e-5 based on the likelihood ratio test were selected.

### Immunocytochemistry

At day 21 of differentiation, human iPSC-derived cardiomyocytes on 13 mm tissue-culture treated coverslips were rinsed with PBS and treated with 100 nM Mitotracker Far Red (Invitrogen P36970) for 35 min at 37°C. Coverslips were fixed in 4% Paraformaldehyde (Electron Microscopy Sciences, 15710) at 4°C for 15 min, rinsed and stored in PBS. Cells were permeabilized with 0.5% Triton-X100 (Millipore 648466) for 10 min at room temperature, rinsed in PBS, blocked in 3% Bovine-Serum Albumin in PBS-Tween 20 0.1% for 90 min at room temperature, and rinsed in PBS. They were incubated overnight at 4 °C in 1:500 Mouse-anti-α-Actinin (Sigma A7811) and 1:300 Rabbit-anti-Pan-Cadherin (Applied Biosystems AB16505) in 3% BSA in PBS-Tween 0.1% at 4°C overnight. Coverslips were washed on a rocker at room temperature 3X for 5 min in PBS-Tween 0.1%, incubated for 1 hour at room temperature in secondary antibodies of 1:400 Donkey-anti-Mouse-488 (Invitrogen A21202) and 1:400 Donkey-anti-Rabbit-568 (Invitrogen A10042), and washed 3X for 5 min at RT on a rocker in PBS-Tween 0.1%. Cells were then stained in 1 μg/ml DAPI for 10 min at room temperature. Coverslips were mounted in Prolong Diamond (Invitrogen P36970) under a #1.5 coverslip. Imaging was done on a Nikon A1R with ×20 objectives.

### Measurement of mitotracker signal

Images were analyzed using Nikon NIS-elements Advanced Research Imaging Software, version 4.30.01. Cell borders were traced guided by the Pan-Cadherin signal and saved as Regions-of-Interest (ROIs). Software-generated measurements of ROI size, equivalent to cell size, and of mean 640 nm signal intensity for each ROI, representing the Mitotracker signal, were exported for analysis.

### Sarcomere classification

Sarcomeres were stained by α-Actinin immunocytochemistry on monolayer iPSC-CMs and analyzed with the following criteria: Class one contained unassembled or newly formed sarcomeres, being sparse, irregular, with gaps, and are not yet directionally organized; class two corresponded to sarcomeres showing higher density and more regular space, but which only just started to be directionally organized; class three contained linear and directionally organized sarcomeres in which Z-disk banding is not yet distinct or predominant, and; class four corresponded to developed sarcomeres with established directionality and running in parallel, with clear banding of Z-disks.

### Quantification and statistical analyses

Results are presented as means ± standard error of the mean (SEM). Statistical testing was performed with Student’s *t*-test, two-way ANOVA with a minimum of three repeats of each experiment. Statistical significance was achieved with a two-sided *p*-value of 0.05. Statistical analyses were performed with GraphPad Prism 8.0.

### Data and code availability

The BioProject accession number for the single-cell transcriptional profiling data reported in this paper is PRJNA817077 (SRA).

## Results

### Single-cell transcriptomic analysis of human iPSCs and derived cardiomyocytes

**FIGURE 1:**
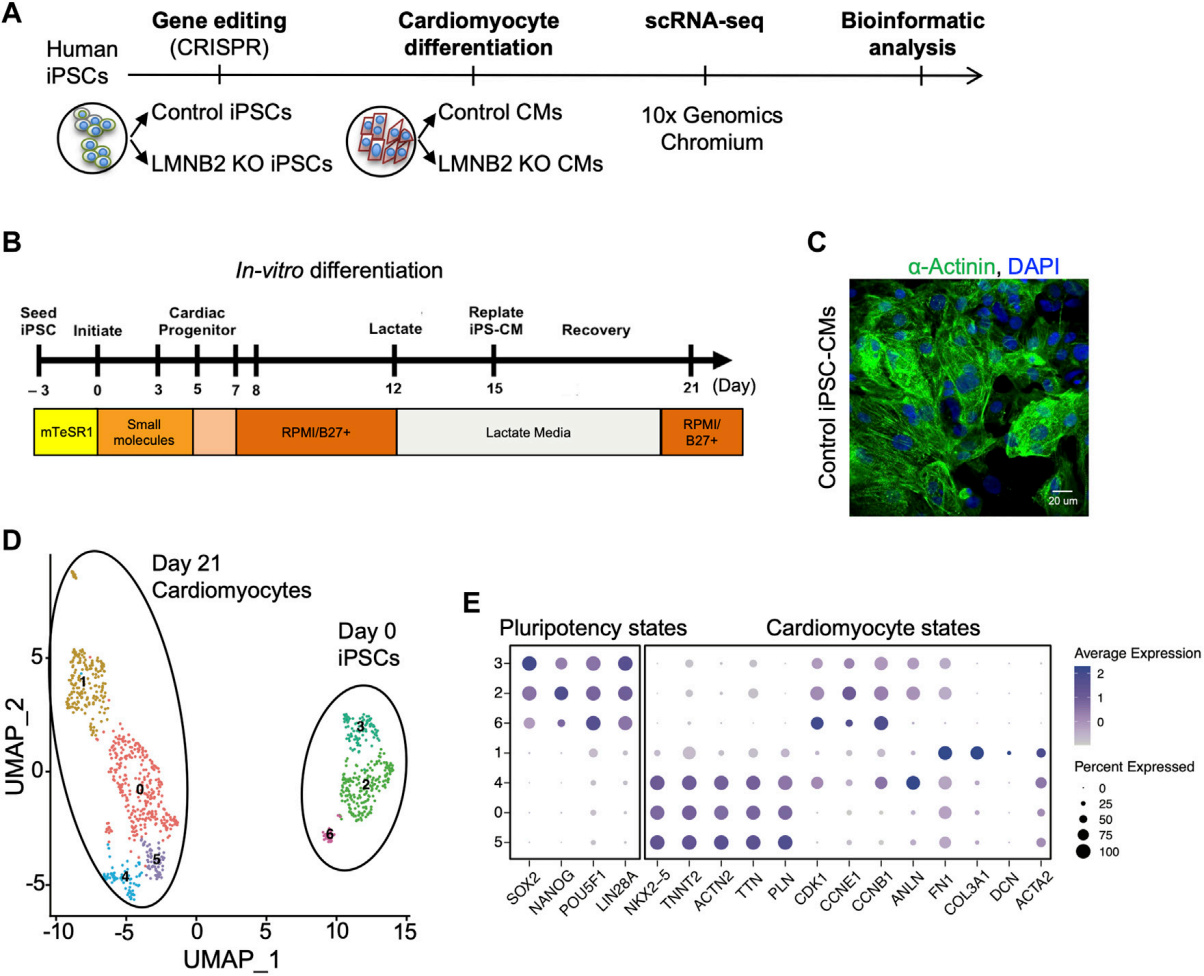
Single-cell transcriptional profiling of human induced pluripotent stem cells (iPSCs) and derived cardiomyocytes. **(A)** Scheme of iPSC-CM generation and single-cell RNA sequencing (scRNAseq). Control human iPSCs (CiPS001-013) and isogenic *LMNB2* KO iPSCs generated by CRISPR-Cas9-mediated genome editing were induced to differentiate into cardiomyocytes by modulation of WNT signaling, followed by scRNAseq at differentiation day 0 and 21. **(B)** Cardiomyocyte Differentiation Timeline: Cardiomyocytes were induced by applying Wnt activator (CHIR99021) and inhibitor (IWR-1) respectively during 0-24 (d0) and 72-120 (d3-d5) hours of the directed differentiation timeline, followed by application of lactate medium at d12 to enrich the cardiomyocyte population. **(C)** Immunofluorescent microscopic analysis of α-Actinin-positive cardiomyocytes at d21. **(D)** Unbiased clustering of scRNAseq results revealed subpopulations of iPSCs (sub-clusters 2,3,6) and cardiomyocytes (sub-clusters 0,1,4,5). **(E)** Dot plots of pluripotency and cardiomyocyte markers in sub-clusters 2,3,6 and 0,1,4,5 confirm identity of iPSCs and CMs populations, respectively.

As the neonatal mammalian heart develops to adulthood *in vivo*, cardiomyocyte maturation increases to culminate in functional optimization. By contrast, although *in vitro* cardiomyocyte differentiation from human iPSCs largely recapitulates cardiomyocyte specification, these cells do not attain maturity (Doss et al., 2012; Lundy et al., 2013; Chong et al., 2014; DeLaughter et al., 2016; Scuderi and Butcher, 2017; Ronaldson-Bouchard et al., 2018). To explore the cellular heterogeneity and to define molecular signatures related to maturation indices within human iPSC-CM populations, we generated single-cell transcriptional profiles of human iPSCs (d0) in comparison with iPSC-CMs collected at d21 (Figure 1A). A high level of purity of iPSC-CMs was achieved by modulating WNT signaling during differentiation days d0-d5, followed by lactate enrichment for cardiomyocytes beginning at d12 (Figure 1B) (Lian et al., 2012; Tohyama et al., 2013). At d21, up to 90% of the cells were cardiomyocytes (Figure 1C). Using a droplet-based 10× Chromium platform, we obtained single-cell transcriptional profiles at day 0 and 21 of differentiation. Unsupervised clustering of single cells at differentiation days 0 and 21 and dimensionality reduction using the Uniform Manifold Approximation and Projection (UMAP) algorithm revealed 7 cell clusters that were grouped into two distinct populations: pluripotent states (clusters 2,3,6) and cardiomyocyte-states (clusters 0,1,4,5) (Figure 1D) (Becht et al., 2018). Among clusters in the cardiomyocyte state, marker gene expression revealed three clusters (0,4,5) that expressed cardiomyocyte transcription factors (*NKX2-5*) and sarcomeres (*TNNT2*), and a single cluster (1) that containing a mixture of fibroblast-like cells, the majority of which expressed the extracellular matrix gene *COL3A1, FN1* (Figure 1E).

### The *LMNB2* gene is not required to maintain pluripotency and proliferation of iPSCs

**FIGURE 2:**
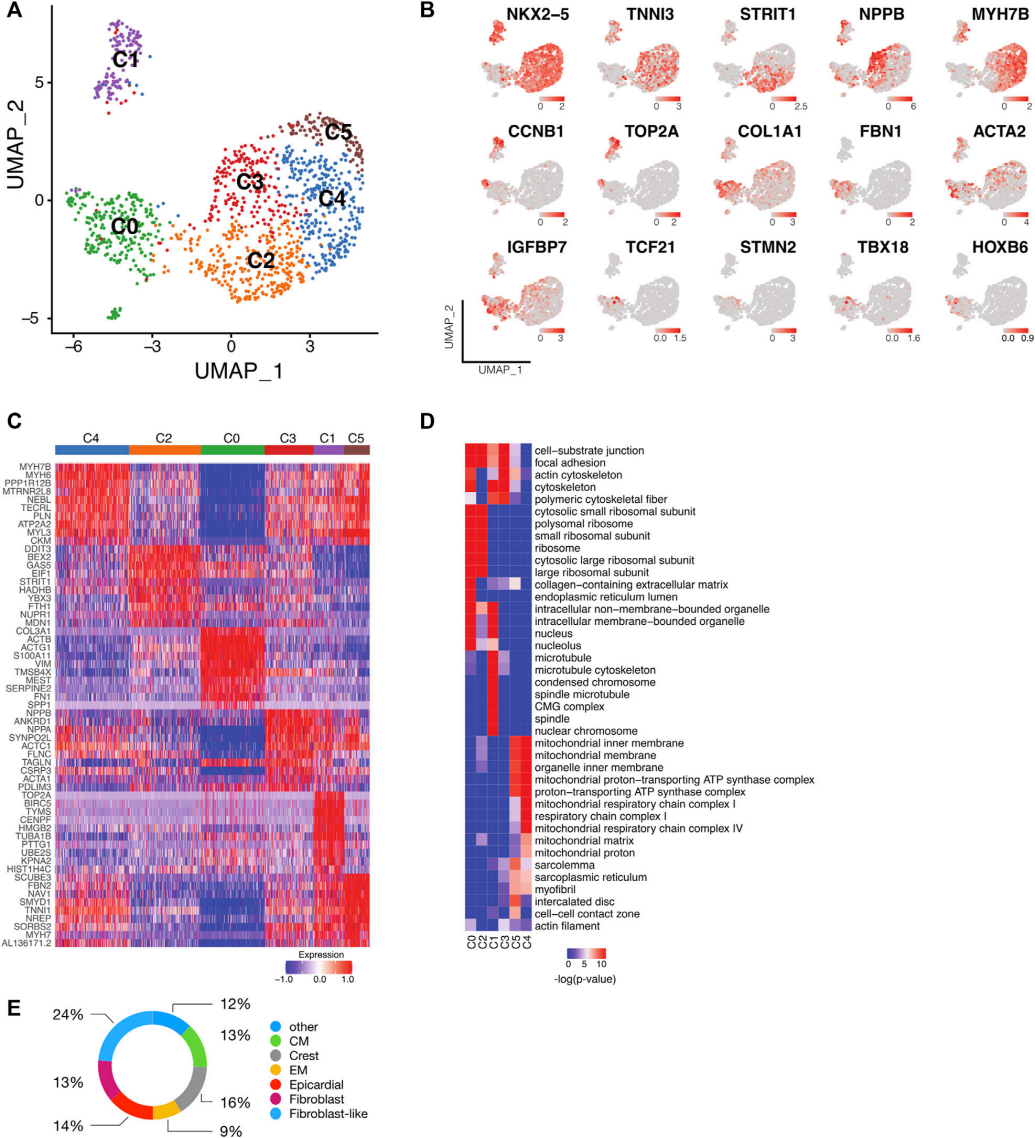
Single-cell transcriptomic analysis identifies subpopulations of human iPSC-CMs. **(A)** UMAP plot of control and LMNB2 KO iPSC-CMs pooled at d21, revealing six major subpopulations. **(B)** Representative gene expression profiles for the six subpopulations shown in A. **(C)** Heatmap showing the top 10 differentially expressed genes within each subpopulation. **(D)** Gene Ontology (GO) analysis of cellular processes associated with each subpopulation. **(E)** Subset analysis of the C0 subpopulation revealing multiple non-cardiomyocyte lineages. CM, cardiomyocytes; Crest, cardiac neural crest/ Schwann progenitor cells; EM, endothelium cells.

In mammals, cardiomyocytes shift their growth models during development. While rapid proliferation and expansion in cell numbers is observed during fetal development, cardiomyocytes gradually exit the cell cycle after birth and switch to cell enlargement accompanied by structural, functional, and metabolic maturation (Chattergoon et al., 2012; Hirose et al., 2019; D'Uva et al., 2015; Soonpaa et al., 1996). During this transition, cell-cycle regulators become repressed as maturation ensues (Soonpaa et al., 1996). However, how proliferation and maturation are intrinsically connected at the molecular level remains unclear. We previously reported that the nuclear lamin gene *LMNB2* is essential for cardiomyocyte division *in vivo* (Han et al., 2020) and in this study we evaluated the role of the *LMNB2* gene in cardiomyocyte maturation when cell division is repressed (Figure 2A). We disrupted the *LMNB2* gene using the guide RNA-directed “clustered regularly interspaced short palindromic repeats” (CRISPR) approach, which generated one-base pair nucleotide insertion in exon 2, thereby creating an isogenic human iPSC line termed *LMNB2* KO iPSC (Supplementary Figure S1A). Knockout of *LMNB2* was validated at the mRNA and protein expression levels (Supplementary Figure S1B, C) and further validation can be found in our previous publication (Han et al., 2020).

Because previous reports described heterogeneous cellular states within human iPSC lines (Nguyen et al., 2018), we performed unsupervised clustering and identified three iPSC subpopulations (C0-2) (Supplementary Figure S1D, E; Supplementary Table S1). Comparison of these three subpopulations in control and *LMNB2* KO iPSCs revealed that *LMNB2* KO iPSCs did not form distinct clusters from control iPSCs (Supplementary Figure S1F). Analysis of the *LMNB2* KO iPSCs indicated that transcripts corresponding to genes encoding pluripotency factors and other nuclear lamins were unaffected **(** Supplementary Table S1 **)**, and we confirmed that both the pluripotency marker Nanog and nuclear lamins were also unchanged at the protein levels (Supplementary Figure S1G, H) (Han et al., 2020). Thus, consistently with a previously published study using a *LMNB2* KO mouse model, our results indicate that the *LMNB2* gene is not required to maintain pluripotency of human iPSCs (Kim et al., 2011). In addition, we performed cell cycle distribution analysis, and observed no significant differences in comparison with controls (Supplementary Figure S2A, B). Furthermore, immunocytochemistry studies with the mitotic marker phosphorylated Histone H3 (H3P^+^) indicated no differences between control and *LMNB2* KO iPSCs (Supplementary Figure S2C). Altogether, our results are consistent with previous findings in mice (Kim et al., 2011) and indicate that the *LMNB2* gene is not required to maintain human pluripotency states nor normal proliferation of iPSCs.

### Cellular heterogeneity arises during cardiomyocyte differentiation

To address cellular heterogeneity, we focused on the transcriptional profiles within clusters corresponding to differentiating cardiomyocytes at d21 differentiation (0,1,4,5; Figure 1E). A total of 1,257 cells from control and *LMNB2* KO groups were captured and profiled. After trimming and filtering, this revealed an average of ∼7,295 genes per cell. Unsupervised clustering yielded 6 cell populations (Figure 2A), among which five (C1-C5) expressed cardiomyocyte markers including *NKX2-5* and *TNNI3* (Figure 2B). Cardiomyocyte population C1 was distinctive in its exclusive content of cycling cardiomyocytes expressing S (*TOP2A*) or G2/M (*CCNB1*) cell-cycle phase genes (Figures 2B,C). Cardiomyocytes in C2 specifically expressed the canonical WNT signaling inhibitor *DDIT3* and genes related to metabolism such as *UNC5B* (Figures 2B,C) (Horndasch et al., 2006). Subpopulation C3 contained cardiomyocytes that specifically expressed *NPPA*, *NPPB* and *ANKRD1*, which are associated with fetal cardiac muscle development and contraction (Figures 2B,C) (Piroddi et al., 2020) and cardiomyocytes in C4 highly expressed *LDB3*, *NEBL*, and *MYH7B* genes, which are involved in cytoskeleton assembly and sarcomere function (Purevjav et al., 2010; Pathak et al., 2021). C5 cardiomyocytes were similar to those in C4, but displayed higher expression of *FBN2, SYMD1,* and *MYH7,* suggesting differences in contractile apparatus assembly and energy metabolism (Figures 2B,C) (Warren et al., 2018; Cui et al., 2019).

We then addressed biological features of the cardiomyocyte populations *via* Gene Ontology (GO) enrichment among the marker genes (Supplementary Table S2). In agreement with the increased cell cycle activity observed in C1 cardiomyocytes, ontology of those cells indicated genes involved in telomere and mitotic spindle function (Figure 2D). Subpopulations C3, C4, and C5 shared similar cellular components but exhibited different functional commitments, e.g., C3 cardiomyocytes were linked to cell-substrate junction and cytoskeletal organization that facilitates muscle cell development, whereas C4 cardiomyocytes showed enrichment in metabolic processes and myofibril contraction (Figure 2D). C5 cardiomyocytes were similar to the C3 and C4 subpopulations, but uniquely enriched in intercalated disk components (Figure 2D). Taken together, these results suggest that cell cycle and maturation signatures, including cytoskeleton assembly, metabolic process, and myofibril organization, are significant determinants contributing to the cellular heterogeneity of human iPSC-CMs.

Although subpopulation C0 was marked by high expression of extracellular matrix genes such as *COL3A1*, only a small subset within the cluster expressed fibroblast marker genes like *FBN1*, suggesting that C0 contained a mixture of different cell types (Figures 2B,C). To further scrutinize C0, this population was compared with an *in vivo* scRNAseq profile described for human fetal heart tissue (Asp et al., 2019; Cui et al., 2019). This comparison revealed that C0 contained myocardial cells including smooth muscle/fibroblast-like cells (37%, expressing *ACTA2, BGN, FBN1, COL1A1*), epicardial and derived cells (14%, expressing *TCF21*, *WT1*, *TBX18*), as well as cardiomyocytes (13%, expressing *NKX2-5*). In addition, cardiac neural crest cells (16%, expressing *STMN2*), endothelium cells (9%), and 12% of the cells that did not match to one specific cell type were detected (Figure 2E).

### *LMNB2* is a key regulator of the proliferation-to-maturation transition in cardiomyocytes

**FIGURE 3:**
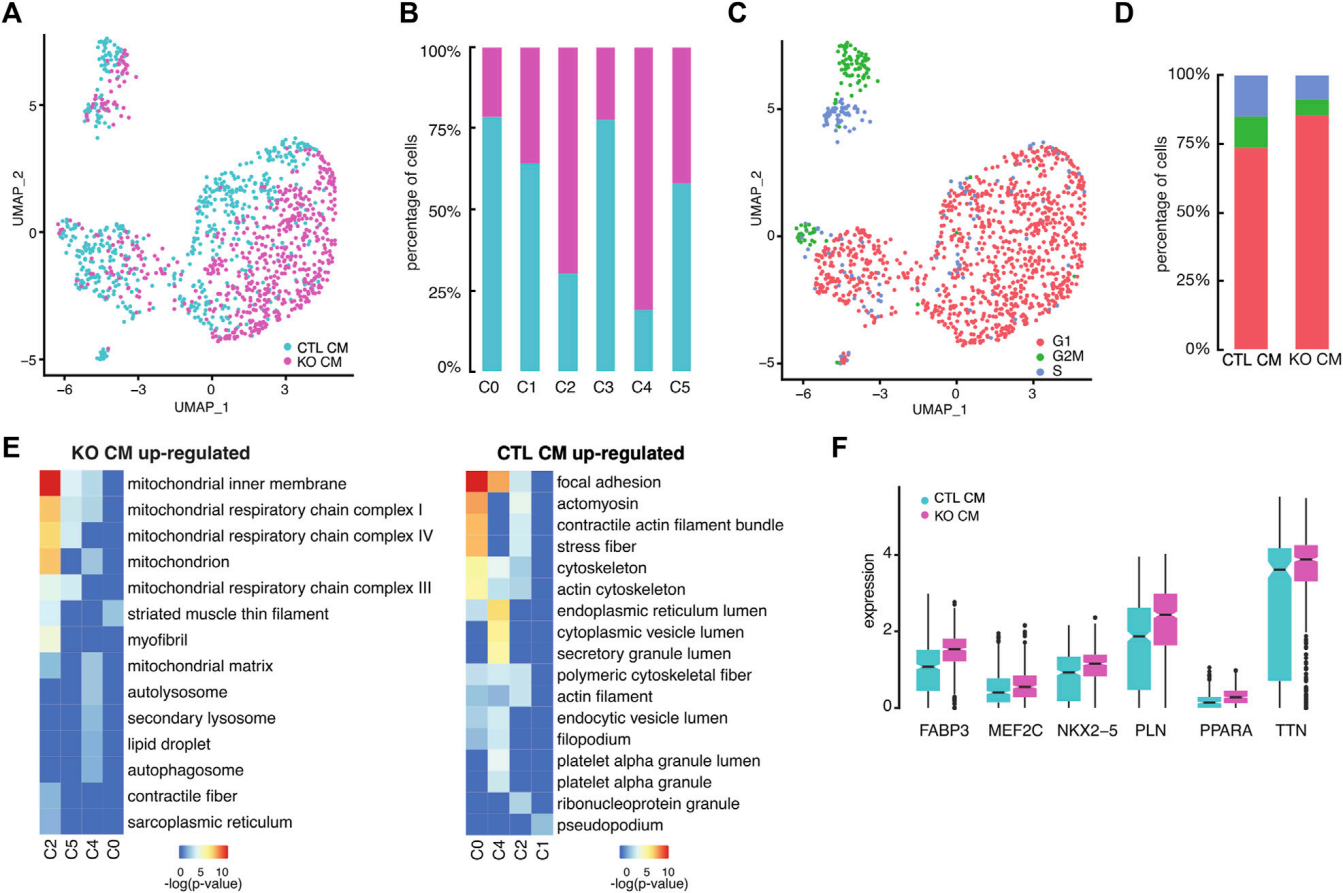
*LMNB2* KO cardiomyocytes exhibit gene profiles indicative of decreased proliferation and increased contractile and metabolic signaling. **(A)** Control and *LMNB2* KO cardiomyocytes identified by UMAP, corresponding to the UMAP clusters in Figure 2A. **(B)** Distribution of control and *LMNB2* KO cells in each subpopulation C0-C5 corresponding to Figure 2A. **(C)** Cell cycle analysis of iPSC-CMs. C1 is comprised of S and G2/M cardiomyocytes. **(D)** Percentages of control and *LMNB2* KO cardiomyocytes in cell-cycle phases G1, S, and G2/M. **(E)** Gene Ontology (GO) analysis revealing top categories within control or *LMNB2* KO in each subpopulation. **(F)** Representative DEGs in control and *LMNB2* KO cardiomyocytes.

*LMNB2* encodes the nuclear lamina protein lamin B2, whose expression is highly abundant in embryogenesis and then undergoes a gradual decrease during postnatal development (Kim et al., 2011). Our previous work identified *LMNB2* as a key regulator of mitotic progression in both mouse and human cardiomyocytes *in vivo* (Han et al., 2020). We therefore aimed to understand the transcriptional consequences and effects on cell cycle status driven by *LMNB2* gene inactivation during cardiomyocyte differentiation. At the single cell level, the relative proportions of control and *LMNB2* KO cardiomyocytes at d21 varied within specific clusters: while clusters C2 and C4 consisted primarily of *LMNB2* KO cells, C0 and C3 had higher proportion of wild type cells (Figures 3A,B). In particular, the percentage of *LMNB2* KO decreased in C0 (36% KO vs. 64% control) and C3 (23% KO vs 77% control) subpopulations and increased in C2 (70% KO vs 30% control) and C4 (81% KO vs 19% control) subpopulations (Figures 3A,B). In the mixed subpopulation C0, the percentage of *LMNB2* KO also decreased (22% KO vs 78% control). Except in cluster C1, the majority of both control and KO cells were non-cycling and at the G1 stage of the cell cycle (Figure 3C). Using the cell cycle prediction analysis, we analyzed 97 cell cycle genes governing S (DNA replication) and G2/M phases and found two distinct subpopulations within C1 that represented S and G2/M respectively (Figure 3C). Overall, *LMNB2* KO cardiomyocytes showed a 28% decrease in both the S phase and the G2/M subpopulations (Figure 3D), which was indicative of an essential function of the *LMNB2* gene in cardiomyocyte proliferation. By contrast, *LMNB2* gene inactivation did not alter the expression of genes that regulate proliferation in human iPSCs (Supplementary Figure S2A, B). We then performed the Gene Ontology (GO) analysis of Differentially Expressed Genes (DEGs) between control and KO in each subpopulation (Figure 3E; Supplementary Table S3) and found that the cellular processes related to mitochondrial function and muscle contractility were upregulated in *LMNB2* KO cardiomyocytes, while cytoskeleton assembly and membrane secretion genes were upregulated in control cardiomyocytes (Figure 3E). A few representative genes encoding pan-cardiac transcription factors (*NKX2-5*, *MEF2C*), muscle development and contractility (*TTN, PLN*), and mitochondrial fatty acid oxidation (*FABP3*, *PPARA*), were also increased in *LMNB2* KO cardiomyocytes (Figure 3F).

### Transcriptional dynamics analysis reveals cardiomyocyte maturation paths

**FIGURE 4:**
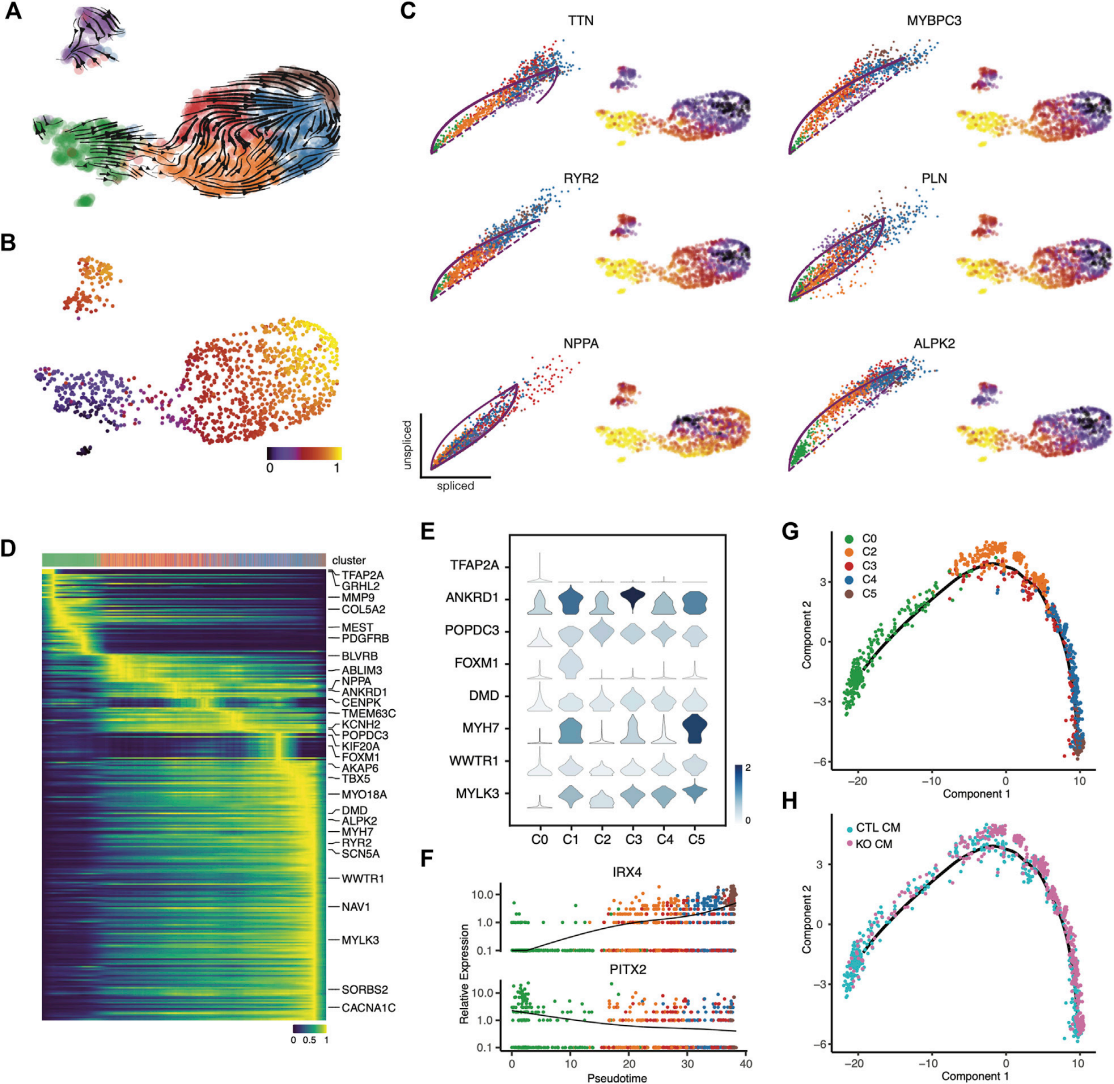
Temporal gene profiles characteristic of cardiomyocyte maturation. **(A,B)** Transcriptional dynamics in cardiomyocyte subpopulations are revealed by RNA velocity analysis. **(C)** RNA velocity of cardiomyocyte maturation-related genes validated the maturation dynamics. (**D**) Top 300 likelihood genes that predicted the dynamical model. The top bar annotates single cells from each cluster along pseudotime stages. (**E**) Representative genes depicting single-cell gene expression from each subpopulation. (**F**) Line plots depict the mean expression of representative transcription factors along the pseudotime trajectory. **(G,H)** Monocle pseudotime trajectory depicting cardiomyocyte maturation dynamics. The cell clusters **(G)** and source **(H)** along the trajectory are marked.

To further understand the role of *LMNB2* in cell state progression during cardiomyocyte differentiation *in vitro*, we performed RNA velocity analysis to capture transcriptional dynamics (La Manno et al., 2018). Notably, we observed two shared but different paths in the direction of cardiomyocyte maturation, namely from C2 to either C3 or C4. These paths ultimately merged toward the boundary between C4 and C5, where a majority of cells were *LMNB2* KO (Figure 2A; Figure 4A). We then performed latent time analysis, which confirmed the temporal transcriptional dynamics in maturing cardiomyocytes (Figure 4B). The expression of several genes, including *NPPA, RYR2, PLN, MYBPC3, TTN,* and *ALPK2*, which are known to be specifically related to different stages of cardiomyocyte maturation *in vivo*, demonstrated dynamics of maturation *in vitro* (Figure 4C) (Churko et al., 2018). Velocity analysis also revealed peak expressions of fetal program genes like *NPPA* in the C3 population. Moreover, sarcomeric genes (*MYBPC3, TTN*), calcium handling genes (*RYR2, PLN*) and *WNT* inhibitor *ALPK2* peaked in both C4 and C5 (Figure 4C).

We used a dynamic model to identify gene candidates that are important drivers of cardiomyocyte maturation along the trajectory path (Bergen et al., 2020). This heatmap analysis revealed 300 genes that dominate this trajectory, displaying a continuous transition toward maturity (Figure 4D, Supplementary Table S4). For instance, *TFAP2A*, a transcription factor required for cardiac outflow tract morphogenesis, was identified at the beginning of the trajectory (Figures 4D,E) (Brewer et al., 2002); a set of genes related to muscle stretch (*ANKRD1*, *NPPA*) peaked during early stages, and peaks of gene expression encoding cAMP binding (*POPDC3*) and ion channels (*SLC1A3, KCNH2*) were observed later in the trajectory (Figures 4D,E) (Froese et al., 2012; Zhong et al., 2015). At the late stage, genes associated with cardiac muscle contraction (*RYR2, DMD, MYH7*) and membrane depolarization (*SCN5A, CACNA1C*) became highly expressed, suggesting increased functional maturation of cardiomyocytes (Figures 4D,E). It is worth noting that genes associated with cell-cycling (*FOXM1*) were expressed during the mid-stages of the trajectory, suggesting a not-fully differentiated state (Figures 4D,E). Furthermore, the expression trends of transcription factors were also consistent with a transition along the maturity path: *PITX2*, a situs-specific transcription factor, showed decreased expression during the trajectory (Figure 4F) (Ryan et al., 1998) but the ventricular transcription factor *IRX4* displayed increased expression (Figure 4F) (Kim et al., 2012). Together with our findings regarding cellular heterogeneity in cardiomyocyte subpopulations, our transcriptional dynamics analysis indicates a pattern of continuous maturation during *in vitro* cardiomyocyte differentiation from iPSCs. These findings also suggest that *LMNB2* gene perturbation promotes cardiomyocyte maturation, and that acceleration of specific gene programs related to energy metabolism and myofibril organization plays a role in the process.

To define the dynamics of temporal gene expression, we performed pseudotime trajectory analysis to reveal the developmental progression of maturation-related genetic programs in iPSC-CMs. We found that cardiomyocytes undergo a continual transition, starting at C0 and ending at C4 and C5 (Figure 4G). This result confirmed the latent time analysis (Figure 4B). Taken together, these findings indicate developmental maturation along the iPSC-CM differentiation *in vitro*. The majority of *LMNB2* KO cardiomyocytes were observed during late stages of development, suggesting increased maturation (Figure 4H). Based on the pseudotime trajectory, we identified distinct patterns for three groups of genes (Suppl. Figure 3; Supplementary Table S5): Group one consisted of genes whose expression increased steadily along the trajectory, the second group included genes that showed a rapid decrease during the early stage of the trajectory and maintained their expression thereafter, and the third group was defined by genes whose expression was repressed at later stages of the trajectory (Supplementary Figure S3). These groups of genes represent potential developmental programs during human iPSC-CM maturation *in vitro*.

### *LMNB2* gene inactivation improves sarcomere organization and increases mitochondrial mass

**FIGURE 5:**
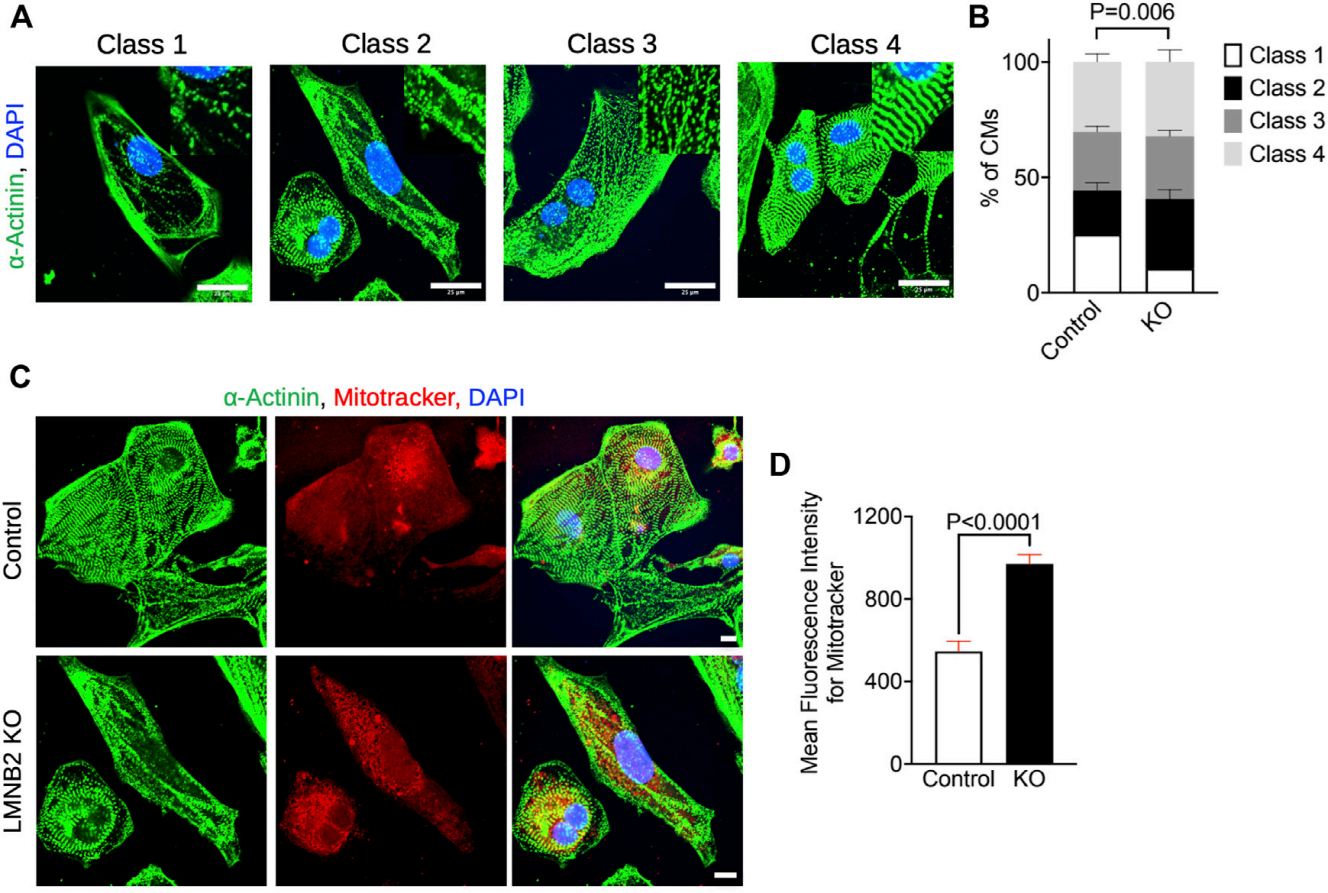
*LMNB2* inactivated iPSC-CMs display increased sarcomere development and mitochondrial mass. **(A,B)** Photomicrographs and quantification of cardiomyocyte sarcomere classification show increased sarcomere development in *LMNB2*-inactivated iPSC-CMs. Control: n = 302 cells, KO: *n* = 312 cells. Four experiments. **(C,D)** Photomicrographs and quantification of mitochondria labeled by Mitotracker-far red show an increase of mitochondrial mass in *LMNB2*-inactivated iPSC-CMs. Control: n = 177 cells, KO: n = 210 cells. Three experiments. Scale bar, 25 µm **(A)**, 10 µm **(C)**. Statistical significance tested with two-way ANOVA **(B)** and unpaired *t*-test **(D)**. Error bars represent mean ± SEM.

To further assess the cellular maturation of control and *LMNB2*-inactivated iPSC-CMs, we performed structural and mitochondrial analysis. We examined the myofibril organization in immunofluorescent images. Sarcomere structure is classified into four categories based on α-Actinin-2 abundance, stripped and parallel structure, and clear banding of Z-disks (Figure 5A). Myofibrils of *LMNB2*-inactivated iPSC-CMs exhibited significantly increased abundance and improved alignment of sarcomeres in comparison with control (Figure 5B), suggesting enhanced structural maturity. As cardiomyocytes mature, mitochondrial size and number increased; assessment by Mitotracker labeling revealed increased mean fluorescent intensity in *LMNB2*-inactivated iPSC-CMs (Figures 5C,D), suggesting an expansion of mitochondria mass. The improved structural and mitochondrial features in *LMNB2*-inactivated iPSC-CMs are consistent with their transcriptional profiles (Figures 3E,F; Figure 4E–H), and support the upregulation of genes involved in fatty acid metabolism (i.e., *FABP3, PPARA*) (Figure 3F). Future experiments will examine how *LMNB2* inactivation affects fatty acid utilization.

### Developmental age of human iPSC-CMs correlates with specific stages of human fetal heart

**FIGURE 6:**
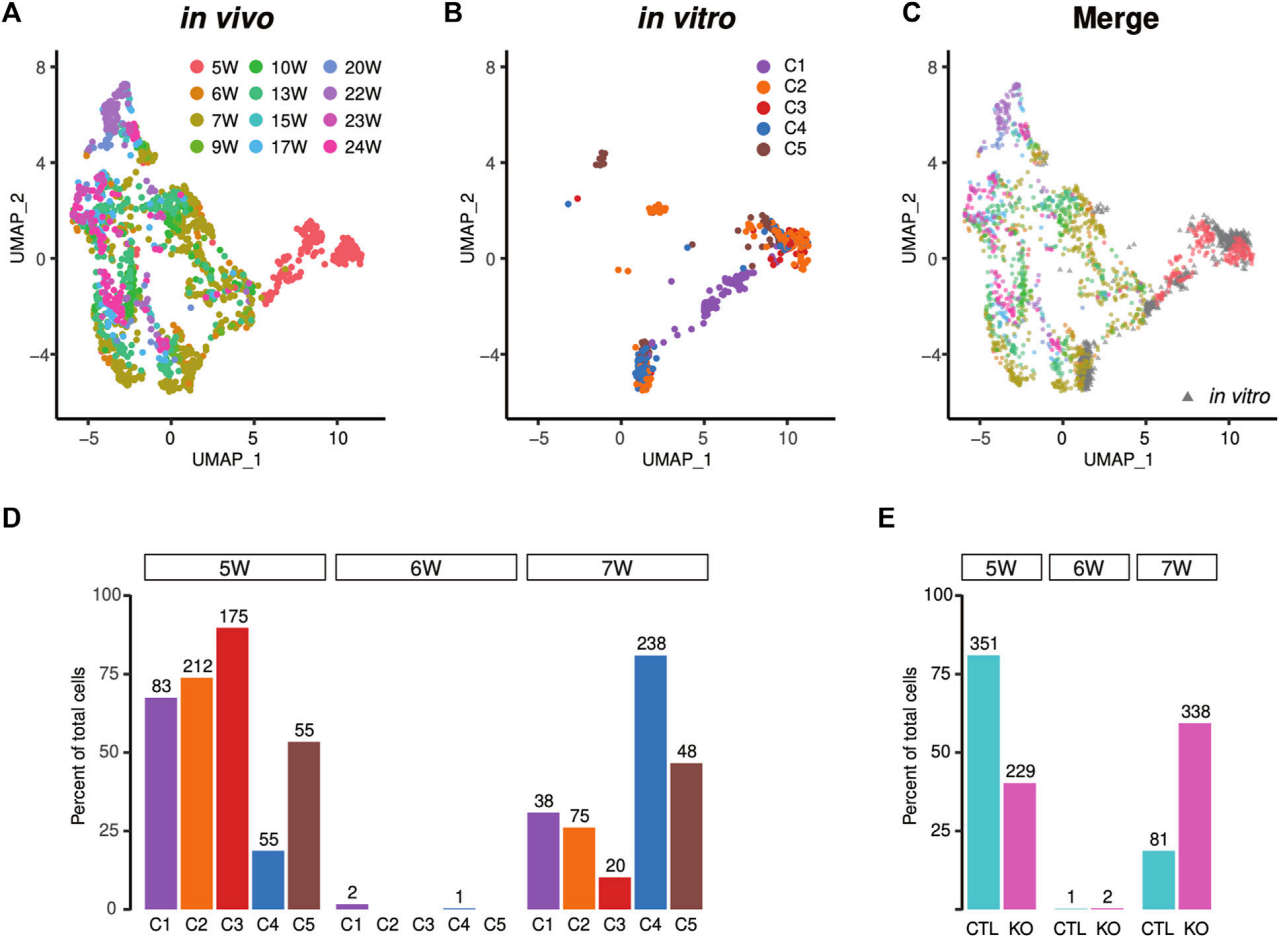
Projection of the single-cell transcriptional profile of *in vivo* human heart tissue reveals increased maturity of *LMNB2* KO cardiomyocytes. **(A–C)** Human iPSC-CMs from control and KO displayed similarity with *in vivo* human cardiomyocytes. UMAP plot of *in vivo* human cardiomyocytes (A and C in color) separating the developmental ages. Human iPSC-CMs (B and C in gray) projected to the UMAP coincided with early *in vivo* cardiomyocytes. The subpopulations (C1-5) in Figure 6B were described in Figure 2. **(D)** Most human iPSC-CMs were similar to fetal cardiomyocytes at 5-weeks’ gestation, while C4 and C5 displayed increased maturity. **(E)** Increased percentage of mature cardiomyocytes derived from *LMNB2* KO iPSCs.

We aimed to determine the developmental age of iPSC-CMs *via* correlation with *in vivo* gene expressions. For this purpose, we compared recently published *in vivo* scRNA-seq data from human fetal heart tissue collected during gestation weeks 5-25 with our scRNA-seq data from iPSC-CMs described in Figure 2. Results of this comparison are shown in Figure 6A (Cui et al., 2019). Both datasets showed high concordance, suggesting that human iPSC-CMs are similar to their *in vivo* counterparts. We found that the majority of human iPSC-CMs mapped to five to seven gestation week heart tissue (Figures 6B,C). Examination of individual subpopulations revealed that approximately 74% (212/287) of C2 and 90% (175/195) of C3 cardiomyocytes corresponded to those in 5-gestation week hearts (Figures 6C,D), while C1 cells corresponded to cycling cardiomyocytes, containing 31% (38/121) of cardiomyocytes that were similar to 7-gestation week heart tissue (Figure 6D)*.* By contrast, subpopulations C4 and C5 displayed improved cardiomyocyte maturity (Figures 6C,D); specifically, 81% (238/293) and 47% (48/103) of cells, respectively in clusters C4 and C5, correlated with 7-gestation week cardiomyocytes (Figures 6C,D). Among the iPSC-CMs corresponding to 7-gestation week heart tissue, 81% were derived from *LMNB2*-KO iPSCs (Figure 6E). Together, our findings suggest that iPSC-derived cardiomyocytes correspond to an early developmental stage of human heart development, and that *LMNB2* depletion increases their maturity.

### Spatial transcriptional analysis reveals subtypes of iPSC-CMs

**FIGURE 7:**
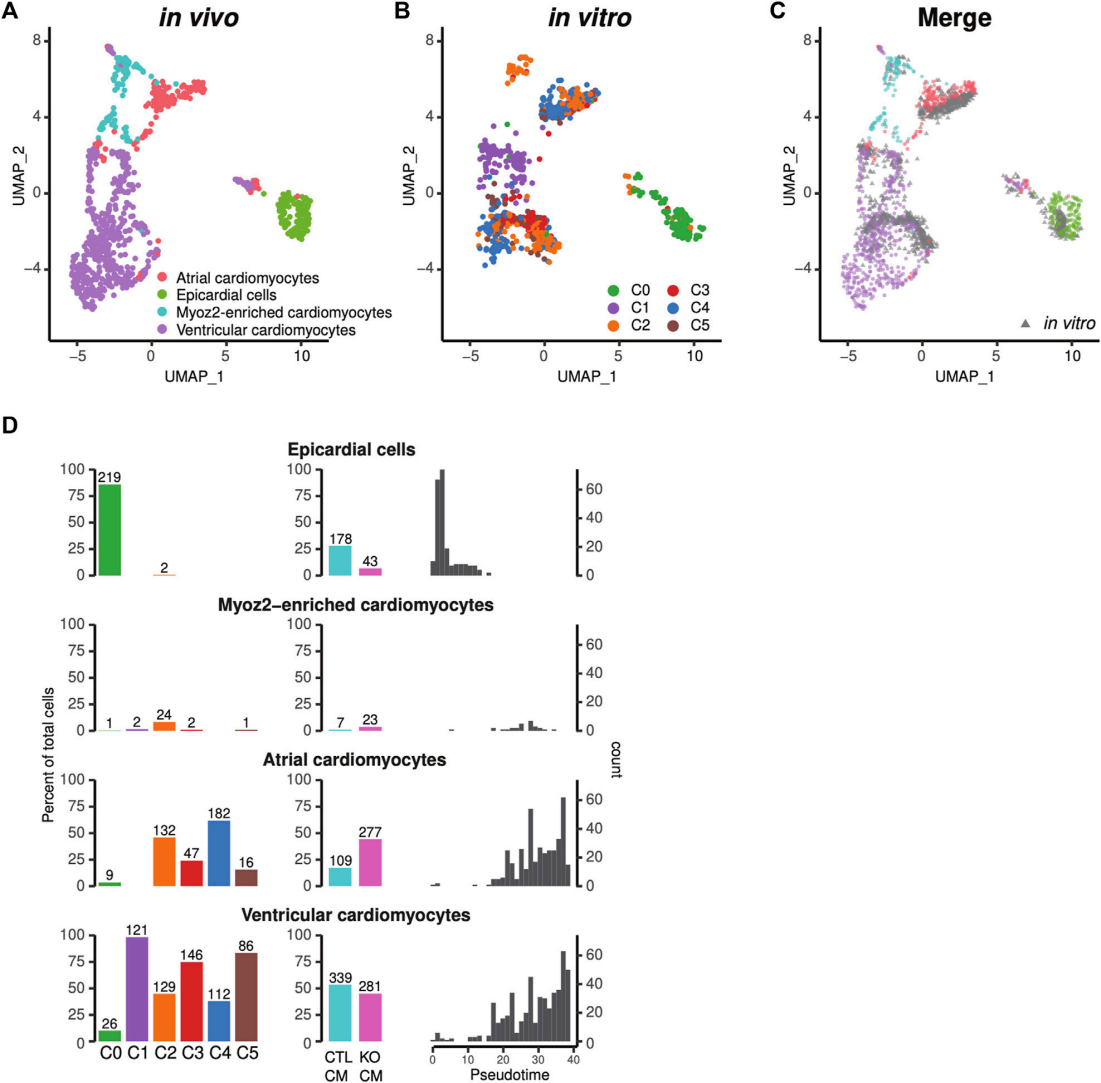
Projection to the single-cell transcriptional profile of *in vivo* human heart tissue revealed different subtypes of iPSC-CMs. **(A–C)** Human iPSC-CMs displayed gene profiles of both atrial and ventricular cardiomyocytes that were similar to *in vivo* human tissue. The six subpopulations in Figure 7B were described in Figure 2. Epicardial cells mapped to the C0 non-cardiomyocyte subpopulation. **(A)** UMAP plot of *in vivo* human heart cells including cardiomyocytes and epicardial cells. **(B)** Human iPSC-CMs projected to the UMAP, and **(C)** overlaid with *in vivo* cardiomyocytes. **(D)** Percentage of epicardial cells, Myoz2-cardiomyocytes, atrial and ventricular cardiomyocytes in each Seurat cluster (C0-C5) (left columns), and in control vs *LMNB2* KO iPSC-CMs (middle columns). Right Column: corresponding developmental ages based on pseudotime analysis.

Human iPSC-CMs are comprised of ventricular-like, atrial-like, and nodal-like cardiomyocytes (van den Berg et al., 2015). We used recently developed spatial sequencing technology to integrate our single-cell RNA profiles of iPSC-CMs (Figures 2A–C) with CM profiles of the embryonic human heart (Asp et al., 2019), and thus to define the subtype heterogeneity of human iPSC-CMs. The majority of human iPSC-CMs corresponded to counterparts within *in vivo* ventricular or atrial anatomical domains (Figures 7A–C) (Asp et al., 2019). A very small fraction was similar to Myoz2-enriched cardiomyocytes, newly defined types of atrial and ventricular cardiomyocytes observed in embryogenesis and adulthood (Figure 7D) (Gladka et al., 2018). The cardiomyocytes in subpopulations C2-5 are mixtures of atrial and ventricular cells, while the cycling cardiomyocytes in C1 are more akin to ventricular cells (Figure 7D). Finally, integrative analysis indicated that the non-cardiomyocyte C0 population was comprised of epicardial and fibroblast/smooth muscle-like cells (Figures 7A–D). Thus, comparison of *in vitro* human iPSC-CMs with *in vivo* cardiomyocyte subtypes revealed characteristics of atrial and ventricular cells consistent with their anatomical localization. *LMNB2-*KO iPSCs generated a higher percentage of CMs with atrial attributes, but similar percentage of ventricular cardiomyocytes in comparison with control (Figure 7D). Notably, when we mapped atrial- and ventricular-like cardiomyocytes to the pseudotime trajectory, we observed continuous maturation of both subtypes (Figure 7D).

## Discussion

Human iPSCs hold great promise for future translational applications, including tissue regeneration and drug screening, as well as to design studies aimed at elucidating the mechanistic bases of disease. However, many lineage-specific cells generated by current differentiation methods are not fully functional (Murry and Keller, 2008). Cardiomyocytes derived from human iPSCs have shown heterogeneous, yet immature phenotypes; therefore, our study sought to identify the transcriptional profiles that define the heterogeneity among human iPSC-CMs, and to further understand the molecular features that define the proliferation-maturation transition, a hallmark in the neonatal phase of heart development. In this study, we described single-cell transcriptomic profiles of human iPSC-CMs. We used the most commonly employed small molecule based WNT signaling modulation method, in combination with lactate treatment, to enrich cardiomyocytes at day 21 of differentiation but chose to increase the sequencing reads of individual cells (average of 248,470 per cell), with an outcome of 7,295 genes on average detected, which allowed us to characterize heterogeneity of these cells with greater detail. Using a mRNA splicing analysis and pseudotime trajectory, we built a developmental path between dynamically transcribed molecules and cardiomyocyte subpopulations. We also identified the maturity state of human iPSC-CMs by comparing our data to a reference map derived from *in vivo* fetal heart tissue.

By leveraging an unsupervised analysis, we were able to identify transcriptional heterogeneity that defined subpopulations of iPSC-CMs. Remarkably, in addition to cell type differences, the heterogeneity among cardiomyocyte subpopulations revealed two important features, namely, cell-cycle and maturity. Consistent with previous reports (DeLaughter et al., 2016; Cui et al., 2019; Li et al., 2019), cycling cardiomyocytes were clustered into a distinct population characterized by the expression of cardiomyocyte marker genes (including *NKX2-5* and *TNNT2*) and cell-cycle marker genes. According to *in vivo* studies, early-stage cardiomyocytes, before E14.5 in mouse and 5-week’ gestation in human, are highly proliferative (DeLaughter et al., 2016; Cui et al., 2019). As genes that activate the cell cycle become inhibited during postnatal stages of heart development, cardiomyocytes become increasingly specialized to optimize contractile function by transcribing genes encoding myofibrillar and energy generating proteins.

We, along with others, have reported that, during *in vivo* heart development, cell-cycle exit proceeds in a stepwise fashion, accompanied by gradual increases in cardiomyocyte polyploidization and maturation until the adult stage is reached (Soonpaa et al., 1996; Murry and Keller, 2008; Bergmann et al., 2009; Mollova et al., 2013; Senyo et al., 2013; Han et al., 2020). To mimic this process in iPSC-CMs, we employed *LMNB2* gene inactivation. Consistent with our previous *in vivo* findings, inactivation of the *LMNB2* gene significantly decreased cell cycle (Figure 3D) and increased polyploidy in cardiomyocytes. The non-cycling cardiomyocytes were grouped into four closely related subpopulations that exhibited distinct transcriptional profiles representing continuous maturity. In those non-cycling cardiomyocyte populations, *LMNB2* inactivation increases expression of genes associated with functional maturation from mitochondrial metabolism to myofibril organization (Figure 3), which are also supported by our experimental results (Figure 5). In addition, two independent trajectory analysis also indicate a higher level of maturity in *LMNB2* KO cardiomyocytes (Figure 4). However, the molecular mechanisms that drive mitochondrial and myofibril maturity *via LMNB2* gene inactivation required further investigation. Moreover, whether polyploidy contributes to the functional maturation needs further characterization. Interestingly, a recent work discovered that lamin B2 levels control nuclear pore numbers during cardiomyocyte maturation and subsequently alter nuclear import of signal proteins (Han et al., 2022). It provides a new perspective to further investigate whether the nuclear pore dynamics influence the gene transcription related to proliferation and cellular maturation of cardiomyocytes.

Integration of our dataset with scRNA-seq data from human fetal heart tissue revealed that iPSC-CMs mapped to fetal *in vivo* counterparts at high levels of similarity, a result that was anticipated because CMs in both sources are immature. However, although 81% of control iPSC-CMs corresponded to human fetal cells at week five of gestation, 60% of *LMNB2* KO cardiomyocytes correlated with cells at later stages of fetal maturation *in vivo*. These results are suggestive of two opposing processes for which a balance must be achieved during advancement to adult stages, namely proliferation *versus* maturation. Previous studies using single-cell RNA-seq reported increased maturation of iPSC-CMs *via* strategies including prolonged *in vitro* differentiation, multilineage organoid culture, activation of hypertrophic signaling, and increased fatty acid metabolism (DeLaughter et al., 2016; Churko et al., 2018; Friedman et al., 2018; Biendarra-Tiegs et al., 2019; Funakoshi et al., 2021; Grancharova et al., 2021; Silva et al., 2021; Wickramasinghe et al., 2022). It is well established that iPSC-CMs are highly proliferative compared to their *in vivo* counterparts. Leveraging our recent mechanistic discovery that *LMNB2* disruption inhibits cardiomyocyte mitotic progression, the findings reported here identified a direct link between cardiomyocyte proliferation and maturation *via LMNB2*. This work points to a possible new strategy for inducing cardiomyocyte maturation.

It is worthwhile to reiterate the specific function of B-type lamins in differentiation and tissue organization. Using single-cell RNAseq, we have confirmed that *LMNB2* is not required to maintain pluripotency and iPSC proliferation. Interestingly, *LMNB2* has been shown to play an essential role in mitosis and differentiation in neurons and cardiomyocytes (Coffinier et al., 2011; Kim et al., 2011; Gigante et al., 2017; Han et al., 2020). However, one discrepancy worth noting is the effect of inactivating the *LMNB2* gene on the cell cycle in iPSC-CMs *versus* CMs in the *in vivo* mouse heart. Using cardiomyocyte-specific Cre recombinase (*Myh6-cre* or *Tnnt2-cre*) to inactivate *LMNB2* in the mouse heart, we observed reduced numbers of cardiomyocytes expressing G2/M phase (mitosis) markers, leading to nuclear polyploidization (Han et al., 2020). By contrast, when the *LMNB2* gene was depleted in iPSC-CMs (Figure 3D), G2/M phase as well as S phase cardiomyocytes were reduced. We speculate that the S phase reduction in iPSC-CMs results from the M-phase block that prevents subsequent cycling. Nonetheless, gene inactivation strategies and species differences may be factors contributing to this phenomenon. From a different perspective, this also implies that human iPSC-CMs are still immature and possess potential to undergo multiple cell cycles. This interpretation is consistent with evidence indicating that, in addition to its role organizing the nuclear envelope/lamina, a major function of *LMNB2* is to regulate the mitotic machinery (Tsai et al., 2006).

In conclusion, these findings illustrate the value of using strategies to integrate with and utilize public single-cell and spatial transcriptomic datasets; here, we were able to map subtypes of human iPSC-CMs to developmental ages of human fetal heart tissue, enabling their assignment to defined anatomical positions and correlation with *in-vivo* cell types. Moreover, this study provides a detailed, multifaceted understanding of the cellular heterogeneity within human iPSC-CM populations, as well as the important finding that disruption of the gene encoding *LMNB2* results in dynamic transcriptional alterations indicative of increased iPSC-CM maturity. Continuing efforts to understand developmental biology at the single-cell level will provide a tremendous knowledge base that will facilitate the generation homogeneous populations of mature, tissue-specific cells in the future.

## Data Availability

The datasets presented in this study can be found in online repositories. The names of the repository/repositories and accession number(s) can be found below: https://www.ncbi.nlm.nih.gov/; PRJNA817077”.
