# Supplementary figures and images for: Single-cell transcriptomic profiling reveals specific maturation signatures in human cardiomyocytes derived from LMNB2-inactivated induced pluripotent stem cells
Source: Front Cell Dev Biol. 2022 Nov 28;10:895162. doi: 10.3389/fcell.2022.895162 (PMC9742441; doi:10.3389/fcell.2022.895162)

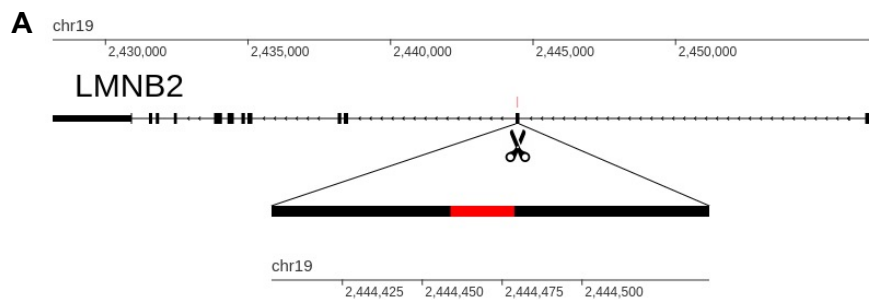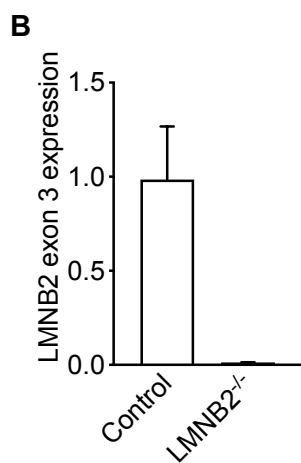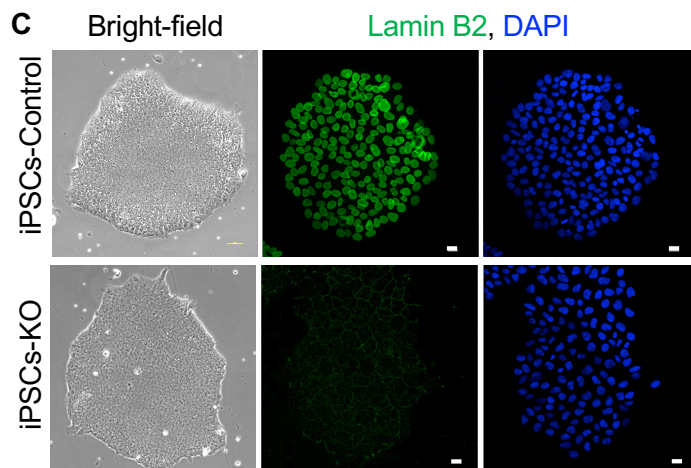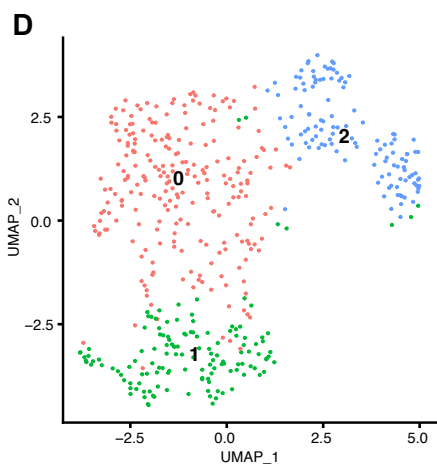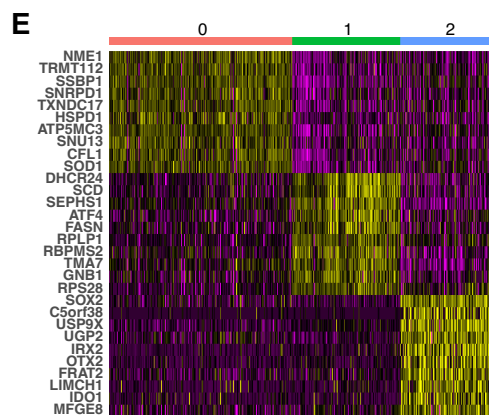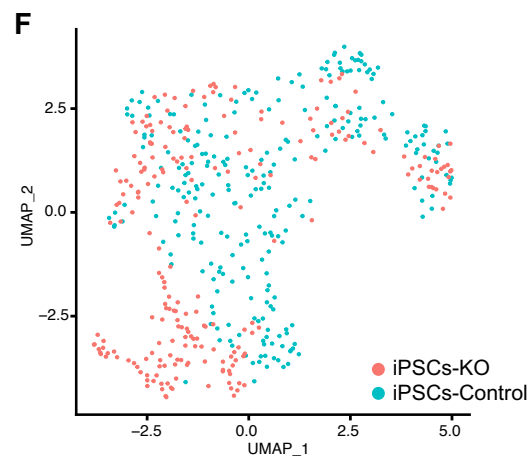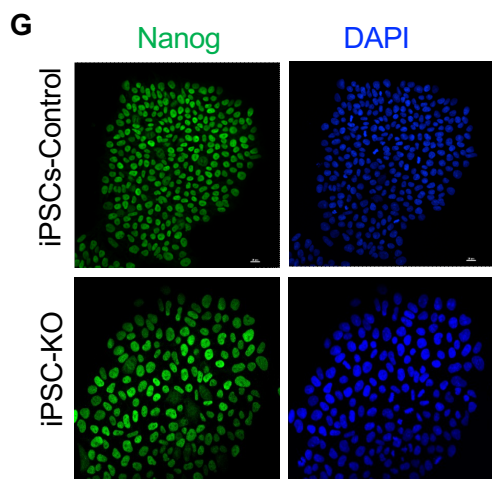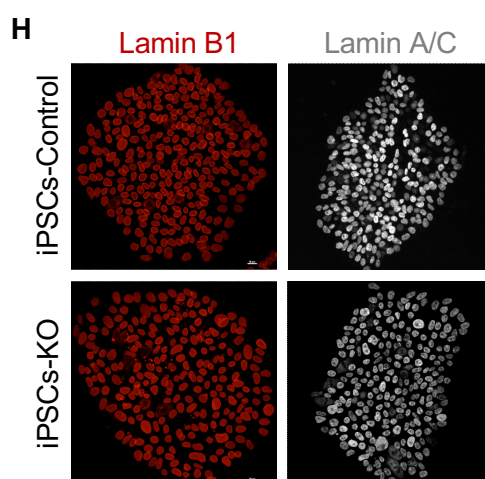

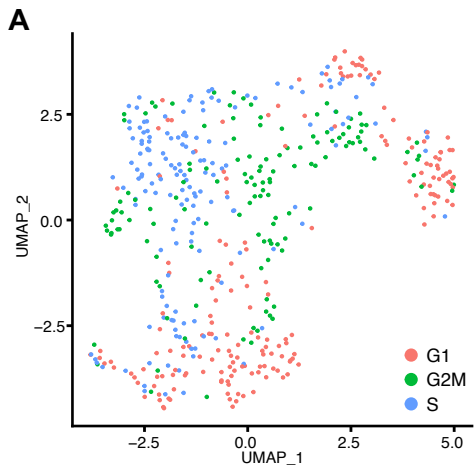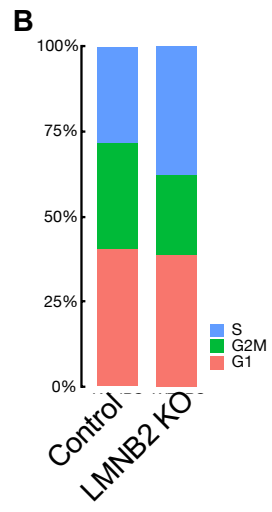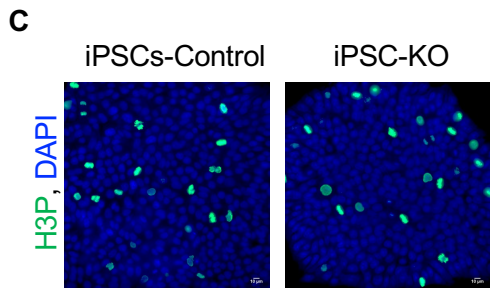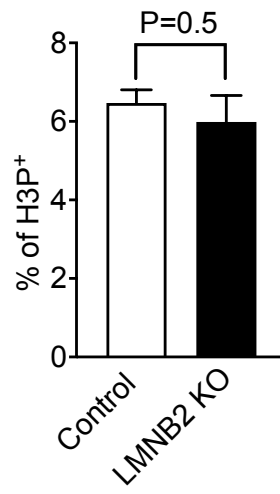

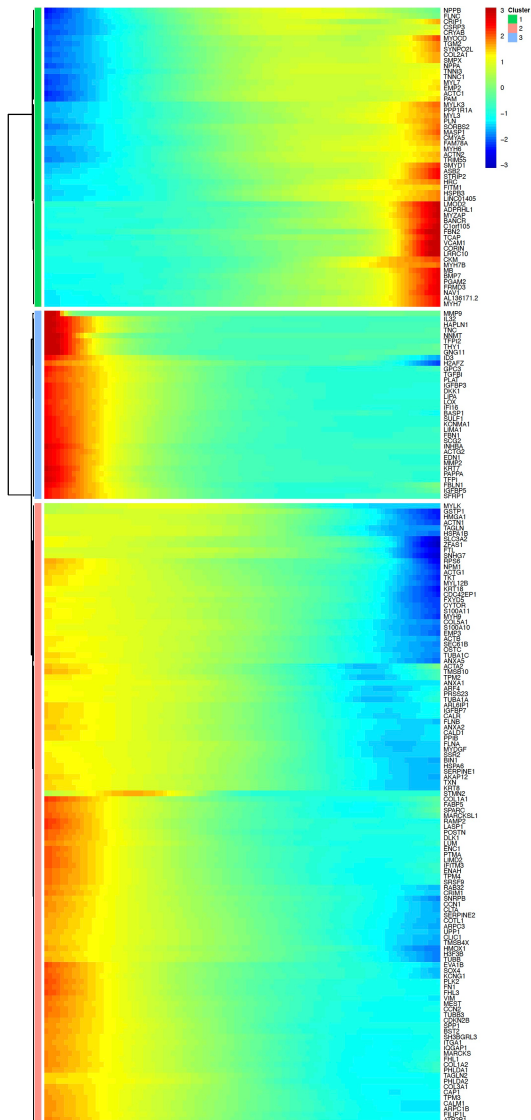

Supplement: Supplementary file 3 [file DataSheet1.PDF]
